# How Morphological Constraints Affect Axonal Polarity in Mouse Neurons

**DOI:** 10.1371/journal.pone.0033623

**Published:** 2012-03-21

**Authors:** Sophie Roth, Mariano Bisbal, Jacques Brocard, Ghislain Bugnicourt, Yasmina Saoudi, Annie Andrieux, Sylvie Gory-Fauré, Catherine Villard

**Affiliations:** 1 Institut Néel and Consortium de Recherche pour l'Emergence des Technologies Avancées, CNRS & Université Joseph Fourier, Grenoble, France; 2 Institut National de la Santé et de la Recherche Médicale, U836-GIN; Commissariat Energie Atomique, iRTSV-GPC, Grenoble, France; University of Antwerp, Belgium

## Abstract

Neuronal differentiation is under the tight control of both biochemical and physical information arising from neighboring cells and micro-environment. Here we wished to assay how external geometrical constraints applied to the cell body and/or the neurites of hippocampal neurons may modulate axonal polarization *in vitro*. Through the use of a panel of non-specific poly-L-lysine micropatterns, we manipulated the neuronal shape. By applying geometrical constraints on the cell body we provided evidence that centrosome location was not predictive of axonal polarization but rather follows axonal fate. When the geometrical constraints were applied to the neurites trajectories we demonstrated that axonal specification was inhibited by curved lines. Altogether these results indicated that intrinsic mechanical tensions occur during neuritic growth and that maximal tension was developed by the axon and expressed on straight trajectories. The strong inhibitory effect of curved lines on axon specification was further demonstrated by their ability to prevent formation of multiple axons normally induced by cytochalasin or taxol treatments. Finally we provided evidence that microtubules were involved in the tension-mediated axonal polarization, acting as curvature sensors during neuronal differentiation. Thus, biomechanics coupled to physical constraints might be the first level of regulation during neuronal development, primary to biochemical and guidance regulations.

## Introduction

*In vivo*, the behavior of cells and tissues is determined by a combination of biochemical and physical signals from the microenvironment. Cells exert forces and sense the environment to modulate their fundamental functions such as migration and differentiation. The impact of the mechanical and geometrical features of the surrounding matrix on the structure and functions of cells has been increasingly documented [1], [2], [3]. In neurons, cytomechanics act at several steps of the developmental program. The balance between proliferation and differentiation of neuronal stem cells is modulated by differential forces [4], newborn neurons are subjected to passive and active mechanical stress that regulates neurite outgrowth and morphogenesis [5], and growth cones pull and stretch neurites [6]. The topology of the environment is crucial during neurodevelopment, as either glial cells bodies or pre-existing axons are physical supports along which neurons migrate or extend axons toward their distant targets [7], [8], [9], [10]. During neuronal differentiation, the nascent axons have to sense and to adapt to the complex topologies arising from the crowded environment of developing brain [8]. How physical constraints of the micro-environment may affect axonal polarization remained poorly described [11], [12]. It is known, however, that submitting equivalent neurites to external tension forces allowed the specification of the stretched neurite into an axon, even in already polarized neurons [13]. At the subcellular level, both neuronal differentiation and the establishment of forces involve cytoskeletal components; axonal specification correlates with cytoskeletal rearrangements, including local dynamic instability of actin and stabilization of microtubules [14]. Also, the crucial contribution of the centrosome as a microtubule-organizing center during axonal specification remains debated. Centrosome location has been reported as a predictor of axonal fate [15], [16], but this capacity was later questioned by both *in vitro* and *in vivo* observations [17], [18].

In this study, we wished to model the physical constraints encountered by differentiating neurons *in vivo*, *e.g.* pre-existing axons or cell bodies, and assess their influence on axonal specification. We thus manipulated neuronal shape through non-specific poly-L-lysine-covered micropatterns [19]. By applying geometrical constraints on the cell body we provided evidence that centrosome location was not predictive of axonal polarization; rather, it responded to axonal location. Then, by varying the directions of neuritic growth, we showed that axonal specification may result from achievement of the highest mechanical tension. More, we demonstrated that axonal specification of neurites grown on curved lines was inhibited. This inhibitory effect toward axon formation was strong enough to counteract the multiple-axon-promoting action of taxol or cytochalasin. Finally, using cytoskeleton-related drugs, we found that microtubules seemed to act as major players in tension-mediated neuronal polarization.

## Results

To assay the effects of physical constraints on neuronal polarization we provided micropatterned substrates to hippocampal neurons in culture, thereby constraining cell bodies and/or neurites. Through photolithography techniques, poly-L-lysine adhesive patterns were engineered on hydrophobic glass coverslips, thus providing adhesive and non adhesive surfaces (Fig. S1A–C) to shape embryonic hippocampal mouse neurons in culture.

**Figure 1:**
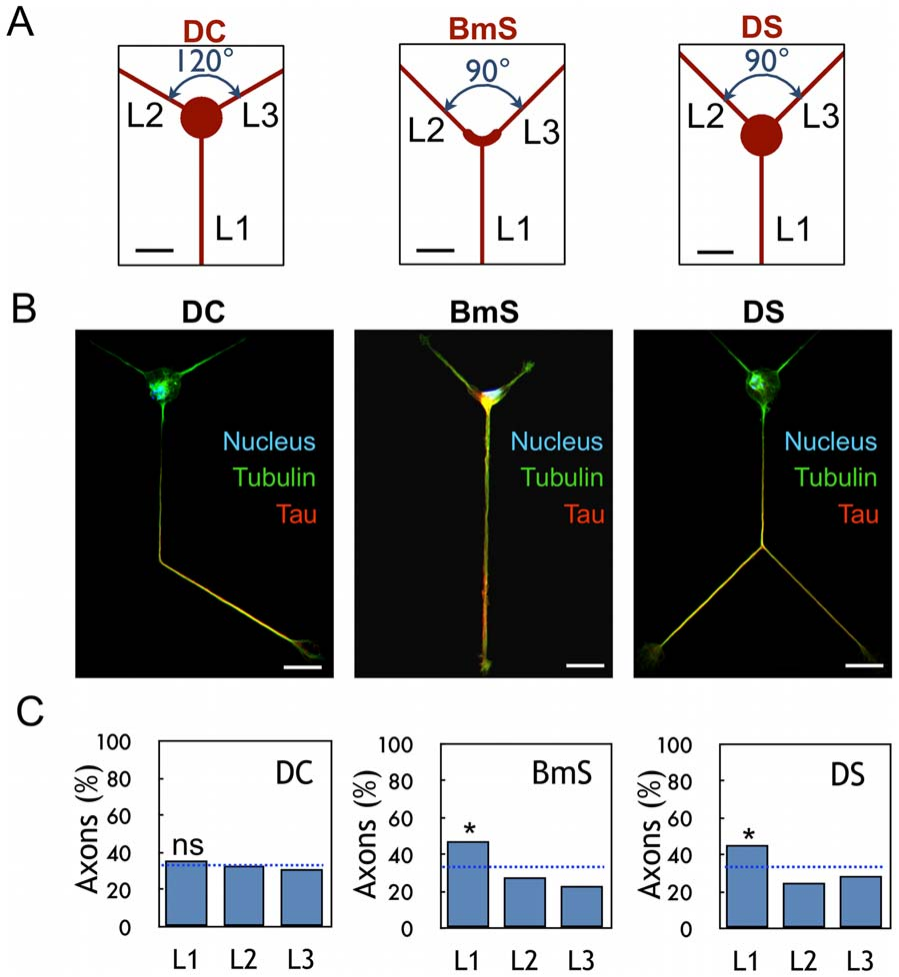
Effect of soma constraints on axonal polarization. (A) Design of patterns DC, BmS, and DS; L1–L3 directions are indicated. (B) Immunolabelings of stage 3 neurons on DC, BmS and DS patterns: axon (tau staining, red), microtubules (tubulin staining, green) and nuclei (Hoechst staining, blue). The shape of the cells reflects the global organization of DC/DS patterns in a hexagonal network. Scale bar, 20 µm. (C) Results of axonal polarization, *i.e.* percentages of stage 3 neurons with their axon along L1–L3 directions (*n* = 194, 176 and 267 for the DC, BmS and DS patterns, respectively). *, significantly different from random (blue dotted line, 33.3% in each direction), *p*<0.05.

A control motif DC, formed with a 20 µm-diameter disk for the cell body and three straight lines (L1–L3 directions) was first built according to a three-fold rotational symmetry (angles = 120°, Fig. 1A). Following neuron plating, we assayed neuronal differentiation after several days of differentiation *in vitro* (DIV). Neurons grown on these micropatterns behaved like freely differentiating neurons [20]: they generated several equivalent neurites after 12 hours (stage 2) and, about 36 hours later, a single neurite underwent rapid elongation and became the axon (stage 3). Accordingly, the early axonal marker tau was found only in the axonal shaft (Fig. 1B). Axonal neurites were also identified using ankyrin G-labelling of the initial segment [21] (Fig. S1D) and dendrites using MAP2 labeling (Fig. S1E).

The percentage of neurons polarized in each direction (L1–L3) was determined and we found random polarization along L1–L3 (35.8%, 33.2%, and 31.1% along directions L1, L2, and L3, respectively, Fig. 1C) as expected from the three-fold symmetry of the DC motif.

Starting from the control DC pattern, new patterns were engineered to analyze relationship between axonal specification and external physical constraints. Geometrical constraints were applied that affected the shape and the surface available for cell spreading and the direction and the trajectories available for neuritic outgrowth.

### Axonal differentiation and centrosome location in the presence of physical constraints on the cell body

First, to study the potential role of centrosome location in axonal polarization, we defined a pattern to geometrically constraint the cell body. Indeed, an L-shaped (boomerang) pattern used to constrain Hela cells had been shown to result in stereotyped cell shape with a centrosome location at the corner of the motif [22]. Two patterns were designed (Fig. 1A and Fig. S2), one with a thick boomerang-like shape (BmS) and another built from a 20 µm-diameter disk (DS). Due to its L-shape, the BmS pattern exhibited an asymmetric direction for neurite outgrowth with an angle of 90° between L2 and L3 and of 135° between the other directions (Fig. 1A). This asymmetry for the direction of neurite outgrowth was reproduced in the DS pattern (Fig. 1A).

**Figure 2:**
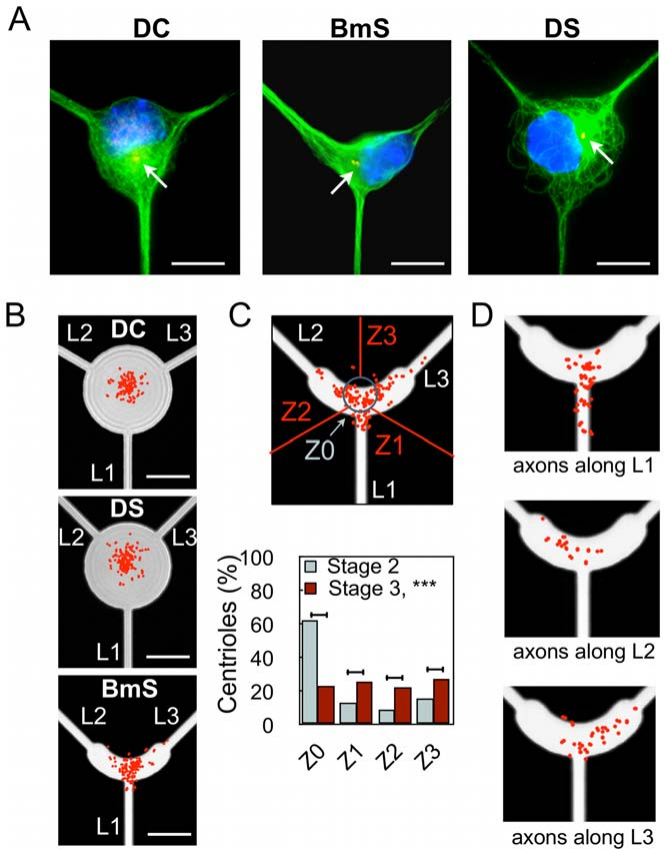
Effect of soma constraints on centrosome position. (A) Microtubule labeling (green), highlighting the different organizations of microtubules in DC, BmS, and DS patterns. Nuclei (blue) and centrioles (red) stained with antibodies against γ-tubulin. Red arrows point to the centrioles. (B) Superimposition of density maps for centrioles and corresponding patterns (*n* = 154, 168, and 160 from stage 2 neurons for the DC, DS, and BmS patterns, respectively). (C) Centrosome distribution in stage 2 (1 DIV) and 3 (3 DIV) neurons grown on BmS patterns. Upper panel: Scheme of BmS pattern indicating the regions of interest Z0-Z3; with the scatter plot of centriole distribution superimposed (red dots, stage 3). Percentages of centrioles in each region of interest with inset showing the density map of the upper scatter plots superimposed on dashed lines delimiting the patterns. (*n* = 160, stage 2 neurons; *n* = 184, stage 3 neurons). (D) Centriole positioning (red dots) and axonal localization in neurons grown over the BmS pattern. (*n* = 31, 12, and 20 neurons for the L1, L2, and L3 directions, respectively).

Centrosome distribution was analyzed from γ-tubulin immunolabelings in stage 2 undifferentiated neurons (1 DIV) (Fig. 2A). The L-shaped pattern BmS was able to induce centrosome distribution along its symmetry axis (Fig. 2B), strikingly reproducing what was observed for HeLa cells [22] and extending to a radically different cellular type the benefits of micropatterns in terms of stereotyped organelle localization. Note that neurons grown over BmS patterns did not display any new actin structures as compared to non-patterned cells *i.e.* stress fibers were not observed (Fig. S3). In contrast, undifferentiated neurons exhibited a largely central centrosome location on DS and DC patterns (Fig. 2B).

On both DS and BmS patterns, axonal polarization was assessed via a positive staining for Tau (Fig. 1B) and preferentially occurred along L1 (44.9% and 47.2%, respectively) as compared to random (Fig. 1C) (*, *p*<0.05) with no significant difference between BmS and DS (*p* = 0.647).

Hence, centrosome location in stage 2 neurons was not predictive of axonal specification at stage 3. After axonal polarization however, centrosome distribution quantified from neurons grown over BmS patterns revealed that, although 64.7% of the centrosomes were still located along the symmetry axis L1 of the pattern, (Z0 area, Fig. 2C, as compared to 87.5% at stage 2 ***, *p*<0.001), the others spread toward L2 (14.1%) and L3 (20.1%) directions. Interestingly, axonal polarization occurred in each direction with the following ratio: 47.2% for L1, 27.8% for L2, and 23.3% for L3 (Fig. 1C) directions. Thus at stage 3, on BmS patterns, the position of centrosomes seemed to be associated with polarization success along each direction. These results indicated a possible redistribution of the centrosome toward the actual axon, following axonal specification. To directly address this possibility we analyzed the centrosome location and the position of the axon from the same individual neurons. As displayed in Figure 2D, location of centrosomes correlated with the direction of axonal specification. Altogether, these results showed that the initial centrosome localization was not the key factor leading to the observed preferential axonal polarization along L1; rather, centrosome position was determined by axonal location.

**Figure 3:**
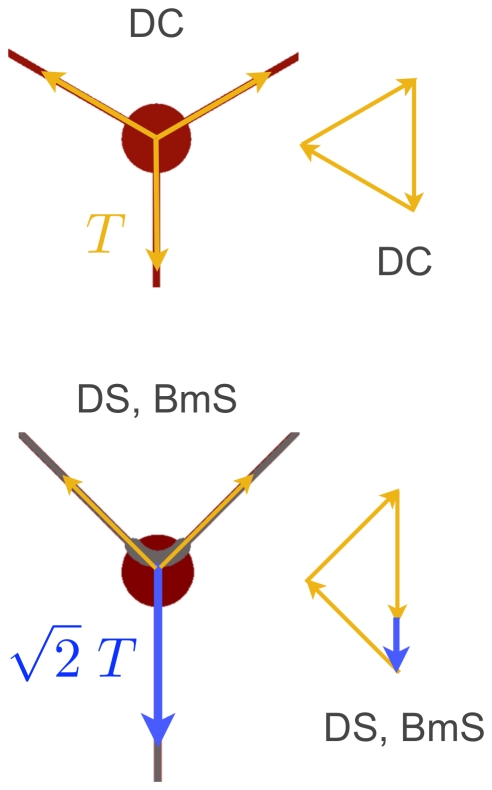
Vectorial analysis of tension forces on DC, DS, BmS patterns. Neuronal directions of outgrowth represented by lines of forces in the DC and BmS/DS patterns. Each vectorial representation shows the magnitude of the tensions (multiple of T, the tension exerted along the L2–L3 directions) exerted along L1 under the hypothesis of a mechanical equilibrium state at the cell level.

To explain the axonal preference along L1 in BmS and DS patterns, we focused on the rotational symmetry breaking in the neuritic directions in these motifs as compared to the DC control pattern. Hence, we considered that neurites were maintained in mechanical equilibrium through the development of mechanical tensions [6], [23]. The vectorial analysis of these tensions yielded different values for their modulus along direction L1 (T_L1_, Fig. 3), *i.e.* T_L1_ was higher by a factor of √2 on DS and BmS than on DC control pattern. This analysis suggested that the neurite that displayed the highest tension probably became the axon and that intrinsic asymmetry of tensions may be involved during axonal differentiation. In brief, intrinsic differential tension was possibly associated with axonal polarization and could trigger a subsequent redistribution of the centrosome population toward the basis of the axon.

### Effect of neuritic constraints on axonal fate

Geometrical constraints were applied to neurite trajectories by imposing curved lines for neuritic outgrowth. By doing so we wished to mimic *in vivo* neuronal path-finding in a crowded environment to determine how the corresponding physical constraints might affect axonal fate.

#### Curved lines for neuritic outgrowth prevented axonal polarization

**Figure 4:**
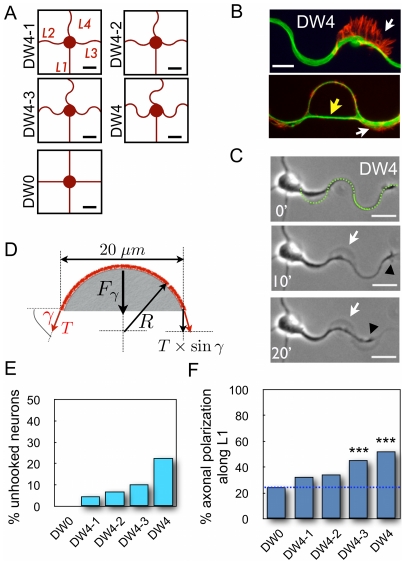
Influence of neurite curvature on axonal polarization. (A) DW4 set of patterns of increasing curvature along directions L2–L4; Scale bars, 20 µm. (B) Partial and complete unhookings observed on fixed cells (microtubules: green, F-actin: red). White arrows point to partial unhooking, characterized by a displaced neuritic shaft still attached to the substrate by a large lamellipodium. The yellow arrow indicates a complete unhooking characterized by a high density of MTs crossing the pattern arch and remaining entities strictly following the curved adhesive line. Scale bar, 10 µm. (C) Time-lapse experiment (indicated in minutes, beginning 30 hours after plating) of a neurite developing on a DW4 pattern showing partial unhooking (white arrow). The black arrowhead points to the neurite tip and the green dashed line marks the position of the adhesive pattern. Scale bars, 20 µm. Refer to Movie S1 for the original time-lapse sequence. (D) Physical modeling of a curved neurite (in red) as an elastic wire under tension Τ. Curvature is reflected by the angle γ (see text), and Fγ = 2 T sin γ (black arrow) is the force experienced by the elastic wire. (E) Percentages of neurons displaying unhookings when grown over DW0 (0%), DW4-1 (4.7%), DW4-2 (7.0%), DW4-3 (10.6%), and DW4 (22.7%) patterns. (*n* = 117, 129, 128, 132, and 132, respectively). (F) Preferential axonal specification along the straight direction L1 were plotted from stage 3 neurons plated over DW0 (24.4%), DW4-1 (32.6%), DW4-1 (34.7%), DW4-3 (45.5%), and DW4 (52.3%) patterns. (*n* = 115, 285, 225, 330, and 216, respectively). ***, significantly different from random, *p*<0.001.

We designed a succession of micropatterns offering a 20 µm-diameter disk (D) dedicated to soma adhesion and 2 µm-thick lines for neurite outgrowth with four directions (L1–L4) made of one straight (L1) and three curved lines (L2–L4) of increasing curvature (Fig. 4A). Curved paths were built from full or truncated half circles of variable radius in order to set the half wavelength of the curvatures to the value of 20 µm (Fig. S4). Additionally, we designed a control pattern named DW0 and characterized by four straight directions L1–L4 (Fig. 4A).

Analysis of axonal specification from 3 DIV neurons grown over this class of micropatterns showed new neuritic outgrowth figures where neurites seemed to be partially (Fig. 4B, upper panel) or totally torn off their curved adhesive track (Fig. 4B, lower panel), which we termed “unhookings”. Video-microscopy analysis of neuronal differentiation showed that neurites dynamically, and sometimes reversibly, unhooked from the curved adhesive track in a time scale of minutes (Fig. 4C, see also Movie S1). Hence, the actual unhookings observed at 3 DIV recapitulated irreversible tearing events that occurred during the first three days in culture. These observations led us to consider the tension forces developed within neurites growing onto curved lines (Fig. 4D). Whenever a neurite undergoes internal tension T, unhooking forces Fγ depending on the specific angle characteristic of each micropattern will tend to tear it off (Fγ = 2Tsinγ, Fig. 4D). Hence, actual unhookings corresponded to neurites whose adhesive forces towards the micropattern were overcome by the unhooking forces when increasing tension developed within neurites. In agreement with the mechanical modelization of Figure 4D, quantification of unhooking events in the different patterns showed that increasing the curvature increased the unhooking events as well, reaching 22.7% of neurons with at least one unhooked neurite on the DW4 micropatterns (Fig. 4E). A possible relationship between unhooking forces and axonal polarization resulted from observations of unhooked neurites. Out of 132 neurons grown over DW4 micropatterns, 30 displayed unhookings (22.7%) unevenly distributed between axonal and non-axonal neurites. Of 67 neurons that polarized along L1, 8 displayed unhookings out of the (3×67) neurites growing on L2–L4, thus indicating a low 4.1% probability of unhooking for non-axonal neurites. In contrast, of 65 neurons with axonal polarization along L2–L4, 18 unhooked axons were counted, indicating a significantly higher 27.7% frequency of unhooked axons (*p*<0.001). Since these results were obtained for DW4 micropatterns with fixed physical parameters (γ = 90°, κ = 0.1 µm^−1^), the different probabilities of unhooking suggest that maximal internal tensions differ for axonal versus non-axonal neurites.

**Figure 5:**
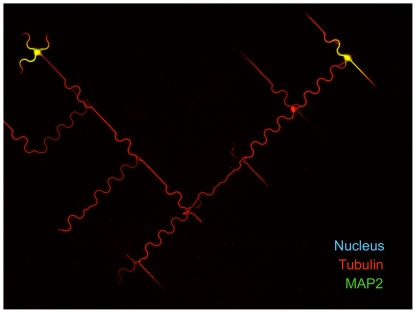
7 DIV neurons spread over DW4 patterns. Dendritic marker MAP2 (green), microtubules (tubulin in red) and nuclei (Hoechst staining, blue). Scale bar = 40 µm. Once formed, the axon developed freely over hundreds of microns along either straight or curved paths following the global organization of DW4 patterns arranged in a network.

Correspondingly, quantification of axonal polarization along each direction showed that axonal polarization along L1 increased with the curvature of the L2–L4 lines (Fig. 4F), reaching 52.3% (*p*<0.001 as compared to random, *i.e.* 25% in each direction) on the DW4 pattern, whereas the other axons differentiated uniformly onto L2–L4 (Fig. S5). We stress here that curvature influenced the process of axonal differentiation but not the process of axonal growth. As illustrated on Fig. 5, once formed, the axon developed freely over hundreds of microns along either straight or curved paths. Taken together, these results indicated that increasing curvature led to increasing unhooking forces, responsible for more actual unhookings and resulting in better axonal polarization along L1, as if curved lines inhibited axonal polarization.

Last, we established a map of centrosome distribution for DW4 micropatterns and verified that centrosome distribution did not predict axonal specification when more directions were provided (4 for DW4 versus 3 for DS) (Fig. S6).

#### Curved lines conflicted multiple-axon-promoting effect of cytoskeleton drugs

**Figure 6:**
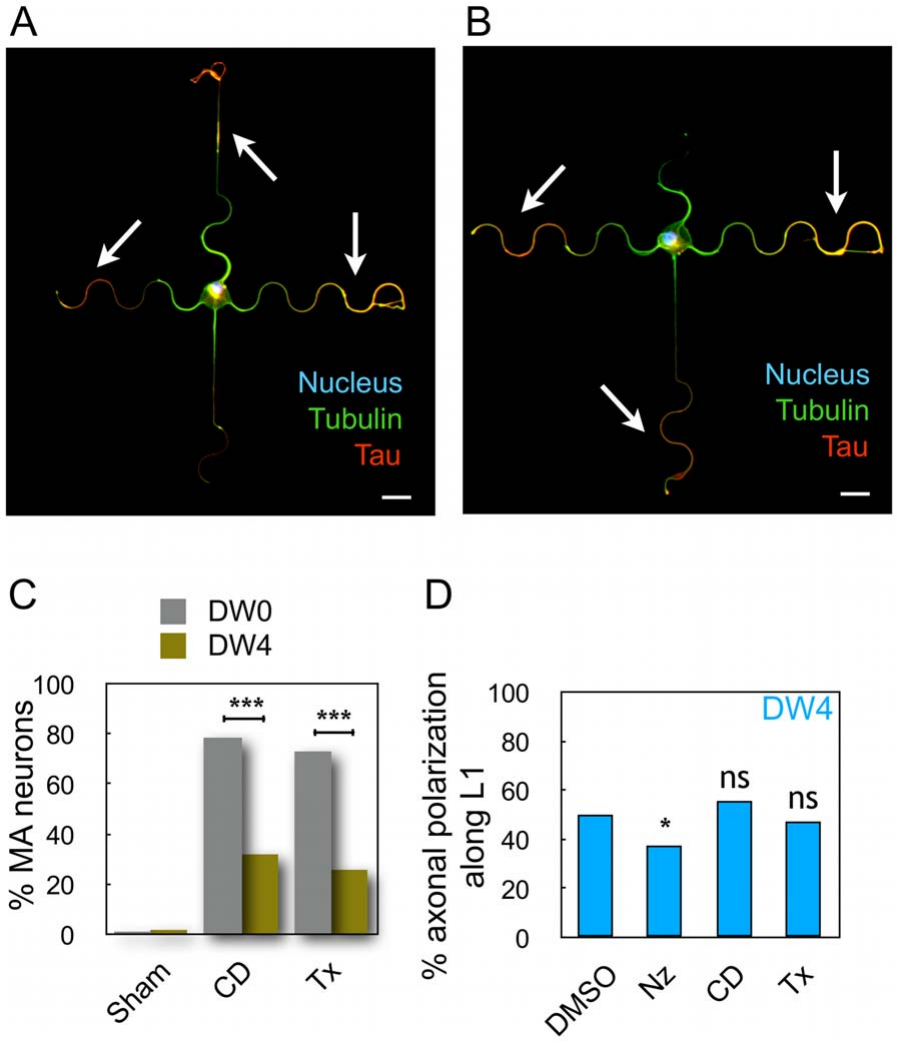
Combined action of drugs and micropatterns on axonal polarization. (A–B) Immunolabeling of stage 3 neurons on DW4 patterns grown in the presence of 0.5 µM cytochalasin D (A) or 3 nM taxol (B): axon (tau staining, red), microtubules (tubulin staining, green) and nuclei (Hoechst staining, blue). Both drugs induced multiple axon (MA) formation as revealed by a tau positive staining of several neurites (white arrows). Scale bar, 20 µm. (C) Percentages of multiple axon (MA) neurons grown over DW0 or DW4 micropatterns, in sham conditions or in the presence of cytochalasin D (0.5 µM) or taxol (3 nM); (sham *n* = 117; CD, *n* = 112; Tx, *n* = 150 for DW0 and sham *n* = 109; CD, *n* = 153; Tx, *n* = 319 for DW4). ***, significantly different from DW0, *p*<0.001. (D) Axonal preference along L1 for neurons grown on DW4 micropatterns, in the presence of DMSO, 45 nM nocodazole (Nz), 0.5 µM cytochalasin D (CD) or 3 nM taxol (Tx) (*n* = 107, 146, 104, and 237 neurons with a unique axon, respectively). Blue dotted lines represent the predicted preference along L1 in the presence of CD or Tx as determined with the probabilistic model (Text S1). *, significantly different from the expected distribution, *p*<0.05.

We further investigated the inhibitory role of curved lines toward axonal polarization by performing experiments in the presence of pharmacological compounds known to promote the formation of multiple axons (MA) in hippocampal neurons grown on flat unconstrained substrates [24], [25]. Neurons grown on DW0 control patterns were treated either with cytochalasin (CD, 0.5 µM), taxol (3 nM), or vehicle. At 2 DIV, the proportion of MA neurons was similar to that reported in the literature, i.e. 78.6% and 73.3% MA neurons in the presence of cytochalasin D and taxol, respectively, while virtually none (1.7%) were observed in sham conditions (Fig. 6).

We then assayed the ability of neurons to develop MA when grown on DW4 patterns. In sham conditions, only few MA neurons were detected (1.8%); in the presence of cytochalasin D or taxol, MA neurons were still observed (Fig. 6A, B) but in significantly smaller proportions than on DW0 (32.0% versus 78.6%, *p*<0.001 and 25.7% versus 73.3%, *p*<0.001 for cytochalasin D and taxol respectively) (Fig. 6 C). These results indicated that curved lines displayed a strong axon-inhibiting effect that opposed the multi-axon-promoting action of the drugs.

#### Microtubules support curvature-mediated inhibition of axonal polarization

The inhibition of axon specification on curved lines most probably involved cytoskeletal relays in neurons. To investigate the involvement of cytoskeleton elements in the inhibitory role of curvature toward axonal polarization, we analyzed axonal preference along L1 from experiments performed in the presence of cytoskeletal-targeted drugs: the actin destabilizer cytochalasin D (CD, 0.5 µM), the microtubule stabilizer taxol (Tx, 3 nM), or the microtubule destabilizer nocodazole (Nz, 45 nM).

Neurons grown on DW4 patterns were treated with each drug and axonal polarization along L1 was measured in neurons displaying a unique axon (Fig. 6D). After nocodazole treatment, as compared to sham conditions, axonal polarization toward L1 was significantly reduced (37.7% versus 50.5%, *p*<0.05), indicating that microtubule integrity was crucial for the inhibitory effect of curved lines toward axonal polarization. On the other hand, cytochalasin and taxol induced the formation of multiple axons and this effect needed to be taken into account: after the differentiation of a first axon, neurons trying to develop a second axon will unequally succeed to do so whether they have developed the first axon on the straight line L1 or along any of the curved lines L2–L4. Thus, the probability of failing to grow a second axon and remaining a neuron with a unique axon will differ according to the position of the first axon. We developed a simple probabilistic model of successful axon specification along straight or curved lines to predict expected values of polarization along L1 in the presence of the multiple-axon-promoting drugs (Text S1). We then compared the predicted values of axonal polarization along L1 with the measured values (Fig. 6D). In the presence of cytochalasin D, the predicted value of polarized neurons in direction L1 was 59.0% and in agreement the measured value was 55.8% (*p* = 0.671, not significantly different). In contrast, in the presence of taxol the predicted value of polarized neurons in direction L1 was 58.4% and the measured value of 47.7% was significantly lower (*p*<0.05).

Altogether these results demonstrated that cytoskeletal elements were differently involved in the inhibitory ability of curved lines to induce axonal polarization. Actin integrity seemed dispensable for the inhibition of axonal polarization by curved lines. In contrast, more neurites grown along curved lines became axons in the presence of taxol or nocodazole *i.e.* curved lines' capacity to inhibit axonal polarization was decreased in the presence of MT-targeting drugs.

## Discussion

### Neuronal polarization is sensitive to external physical constraints

*In vivo*, neuronal differentiation and axonal specification are both under the control of a large number of parameters including adhesion [26], [27] to the extra-cellular matrix, complex responses to guidance molecules [8], and physical constraints [11], [12]. In this study, we analyzed the role of specific physical parameters on axonal specification. We developed a simplified protocol where neurons were plated on top of geometrically constrained micropatterns in a defined cell culture medium. By using various micropatterns, we provided evidence that neuronal polarization was indeed sensitive to external constraints such as curved trajectories for neuritic outgrowth. Our results also indicated that axon polarization was favored along straight lines; such a property might be used *in vivo* by newborn neurons when extending their nascent axon along pre-existing straight structures. Indeed, hippocampal granular neurons extend and fasciculate their axons in the same direction, *e.g.* dentate gyrus neurons extending axons to form the mossy fiber bundle [28].

### Geometrical constraints revealed internal neuritic tension

The involvement of forces during neuronal differentiation was first described for the growth cone of chick sensory neurons and PC12 cells that pulled onto neurites [6], [29]. Mimicking such forces by mechanically pulling a neurite with a micropipette even caused its active growth [29], with constant parameters dependent on intact actin and microtubular networks [30], [31]. Similar experiments with rodent hippocampal neurons unambiguously demonstrated that pulling a neurite could change it into an axon [13]. Finally, identification of low velocity transport independent from the growth cone [32] and observations of axonal stretching from fixed reference points in chick sensory neurons [33] confirmed that internal neuritic tensions may act in living neurons. Our work extends these observations by revealing endogenous neuritic tension in mouse hippocampal neurons grown on micropatterns. In our system, neurons grown over curved lines displayed figures of unhooking formed by neurites progressively detaching from the curved lines they were growing on. This observation led us to consider that individual neurites were submitted to a fine balance of forces, F_adhesion_ and F_unhook_, the latter depending on the curvature of its substrate. Recent modeling of chick sensory neurons estimated the friction coefficient relative to adhesion to be about 9600 N.s.m^−2^ [34]. Such adhesion along a full curved line of the DW4 motif (area = 62.8 µm^2^ for γ = 90°, see Fig. S4) would correspond to a force F_adhesion_ = 1–10 nN to detach in 1–10 min (Fig. 4C and Movie S1). Interestingly, this value of 1–10 nN is of the same order of magnitude as estimations of resting tension in neurites of PC12 cells (1 nN) or Drosophila neurons (4 nN) and of tension needed to differentiate neurites of rat hippocampal neurons into axons (0.4–1 nN) [33], [35], [36].

### Differential internal neuritic tensions may be involved in axonal polarization

The angular orientation applied to straight neuritic directions seemed to be involved in axonal polarization preference (DS micropattern, Fig. 1), suggesting asymmetric internal tensions during axonal differentiation. This observation led us to propose that the neurite expressing the highest tension probably became the axon. Then, the simple mechanical model displayed in Fig. 4D suggested that neuritic tension may be causal in the unhooking phenomenon revealed in neurons grown over the DW class of micropatterns. When unhooking occurred, we very often observed a pause in growth cone advance and even neurite retraction (Fig. 4C, black arrowhead). Such events could result from a collapse of the initially stretched neurite by the disruption of its adhesive contacts with the PLL curved stripe [34]. Therefore, unhookings could participate to inhibiting axonal polarization on curved lines by actively reducing neuritic tension, thereby introducing a differential tension between neurites. Note that curvature itself could also be inhibitory and unhookings mere consequences (see below).

### Centrosome positioning is not predictive of axonal polarization but rather respondent to neuritic tension

The localization of centrosome at stage 2 did not seem to be predictive of axonal polarization, as using BmS and DS patterns resulted in the same polarization ability. More, a significant axonal preference for L1 was observed in DW4 despite the mainly central centrosome localization imposed by this pattern at stage 2. The centrosome has been reported to be highly motile during axonal differentiation [37], [38] and accordingly we found that centrosome distribution changed between stage 2 and stage 3, being clearly aligned along the chosen axonal direction at stage 3. The sensitivity of centrosome positioning to neuritic forces could be further expressed in the course of axonal polarization. At the end of stage 2, one neurite will take precedence over the others and develop a higher force, thus reorienting the centrosome. At this point, the centrosome position may correspond to the consequence of the active cytoplasmic flux toward the most active neurite, *i.e.* the developing axon [18]. It was suggested that centrosome location at the basis of the axon may further stabilize the emerging axon but it was recently shown that centrosome ablation after neuronal specification did not modify axonal growth [39].

### Curvature-mediated inhibition of axonal polarization relies on MT cytoskeleton

On DW4 micropatterns, no change of axonal preference toward the straight direction L1 was observed in the presence of cytochalasin D, indicating that the molecular support of curvature-mediated inhibition of axonal polarization was not strongly affected when the actin network was perturbed. In contrast, treatment with taxol or nocodazole induced less L1 preference than expected from the observations made in the presence of the vehicle. Both drugs are known to affect the microtubule network but their effect strongly depends on the concentrations used in experiments. Low doses of taxol (below 10 nM) affect microtubule dynamics (growing and shortening events at the ends of microtubules) without inducing massive microtubule stabilization and without increasing the microtubule mass [14], [40], [41]; similarly low doses of nocodazole (<100 nM) modulate the dynamics of microtubule without depolymerization [42]. In our study we used such low doses of both taxol (3 nM) and nocodazole (45 nM) to affect microtubule dynamics and we observed decreased axonal preference toward L1. Given our observations of unhooking figures on the one hand and the vectorial analysis of forces (Fig. 3 and S6) on the other hand, it seems that forces mainly express in the axonal shaft. We may speculate that microtubule dynamics in the axonal shaft are linked to neuritic tension: disturbances of microtubule dynamics will affect the mean length of individual MTs within the axonal shaft [43], [44], allowing them for more or less resistance to bending along curved lines. Interestingly, a recent study using rat dorsal root ganglion neurons grown over propylene tubular surfaces demonstrated that curvature *per se* could be used to control the direction of spontaneous neuritic growth [45]. Neuritic outgrowth was inhibited by the curvature of the tubes when it reached values >0.05 µm^−1^, much similar to the curvature of DW4 curved lines (0.1 µm^−1^ when γ = 90°). Using these values, the authors estimated neuritic bending stiffness and indicated that it was compatible with that of bundled MTs [45]. These data, in addition to our results showing that microtubule integrity and dynamics were necessary for axonal polarization, support the hypothesis that MT may be curvature sensors during neuronal differentiation.

## Materials and Methods

### Micro-pattern fabrication

Poly-L-lysine patterns were transferred on glass substrates silanized with 3GPS [46] using UV classical photolithography steps, including Shipley S1805 photoresist spinning (4000 rpm, 0.5 µm thickness, 115°C annealing step for 1 min), insulation through a mask, development (Microposit concentrate 1∶1, Shipley), PLL deposition (1 mg/ml one night), and lift-off using an ultra-sound ethanol bath.

### Neuron culture and labeling

Mouse hippocampal neurons were prepared as previously described [47] and plated at a concentration of 10,000–20,000 cells/cm^2^. For centrosome, tau and MAP2 immunolabelings, neurons were fixed for 30 min in 3.7% formaldehyde/0.5% glutaraldehyde and then permeabilized for 1 min with 0.1% triton ×100. For Ankyrin G immunostaining, neurons at 6–7 DIV were fixed for 6 min in methanol (−20°C). Primary antibodies: mouse mAbs against Ankyrin G (Santa Cruz, Heidelberg, Germany); Tau (clone tau-1, Millipore, Molsheim, France); MAP2 (clone AP-20, Sigma, Lyon, France); rat mAb against tubulin (cloneYL1/2), and rabbit Ab against γ-tubulin (M. Bornens, Institut Curie, Paris, France). Secondary antibodies were Alexa488- or Cy3-coupled (Invitrogen, Villebon-sur-Yvette, France). Isolated neurons were analyzed with an inverted microscope Axioskop 50 (Carl Zeiss, Inc., Le Pecq, France) controlled by Metaview software (Universal Imaging, Downingtown, PA, USA) using a 40× and 63× oil-immersion objective. Images were digitized using a Coolsnap ES camera (Roper Scientific, Trenton, NJ, USA).

### Centrosome analysis

Image sortings were performed using Labview vision software (National Instrument) and a semi-automatic interface that positioned the motifs associated with each pattern. The two centrioles were visible in more than 85% of cases and were then pointed separately. When indistinguishable, the unique fluorescent point counted for two centrioles. Density maps of centrioles' position were achieved by a custom-made Matlab program using an algorithm for smoothing of two-dimensional histograms [48]. The centrosome distribution according to ROIs was assessed using programs in the free Octave language, administered by the GNU General Public License.

### Statistics

All percentage comparisons were performed using χ^2^ tests as implemented in Prism 4.0 (GraphPad Software, La Jolla, USA).

## Supporting Information

Figure S1 **Micropattern with contrasted adhesiveness.** (A) Micrograph of a micropattern showing the mask used during photolithography and properties of the resulting surface: adhesive in white and non adhesive in black. (B) Micrograph of the micropattern showing the adhesive surface covered by FITC-grafted poly-L-lysine (green). (C) Micrograph showing a hippocampal neuron (phalloidin-Texas red staining of actin) spread on the adhesive surface (poly-L-lysine in green). Scale bar 20 µm. (D) Micrographs of hippocampal neurons after 7 days *in vitro*. (Left) axonal labeling with ankyrin G (red), microtubules (tubulin, green) and nuclei (Hoechst staining, blue); (Right) dendritic marker MAP2 (green), microtubules (tubulin, red) and nuclei (Hoechst staining, blue). Scale bar = 20 µm.
(DOC)

Figure S2 **Bm, DC and DS motifs, geometrical details.** (A) Geometrical dimensions of the Bm elementary motif. (B) DC and DS patterns, built with different orientations of the L2–L3 directions.
(DOC)

Figure S3 **Actin network of a stage 2 neuron (1 DIV) grown on BmS pattern.** Cell nucleus (Hoechst staining, blue), centrosome (γ tubulin labeling, green), and actin (phalloidin-Texas red staining, red). No actin stress fibers are visible around the cell body. Scale bar, 10 µm.
(DOC)

Figure S4 **Summary of geometrical characteristics of DW patterns.**
(DOC)

Figure S5 **Influence of neurite curvature on axonal polarization.** Axonal specification along the L1–L4 directions was plotted for DW0 and for each DW4 pattern. The number of neurons polarizing along L1 increased with curvature; other neurons polarized uniformly along L2–L4 directions. ***, significantly different from random distribution (dotted line), *p*<0.001. DW0: 24.4%, 24.4%, 26.0% and 25.2% for L1, L2, L3 and L4, respectively. DW4-1: 32.6%, 24.6%, 21.1% and 21.8% for L1, L2, L3 and L4, respectively. DW4-2: 34.7%, 23.6%, 23.6% and 18.2% for L1, L2, L3 and L4, respectively. DW4-3: 45.5%, 18.2%, 19.7% and 16.7% for L1, L2, L3 and L4, respectively. DW4: 52.3%, 13.3%, 18.8% and 15.1% for L1, L2, L3 and L4, respectively.
(DOC)

Figure S6 **Centrosome distribution and correlation with axonal localization in stages 2 (1 DIV) and 3 (3 DIV) neurons grown over DW4 patterns.** (A) Top: an example of microtubule (green), nuclei (blue) and centrosome (red and red arrow) immunolabelings. Scale bar = 10 µm. Bottom: Superimposition of centrosome density map from stage 2 neurons (*n* = 174) and DW4 micropattern; Scale bar = 10 µm. (B) Top: Scheme of a DW4 pattern and regions of interest Z0–Z4; the scatter plot of centrosome distribution from stage 3 neurons was superimposed (red dots). Bottom: Percentages of centrosomes located in each region of interest from stage 2 and stage 3 neurons (*n* = 174 and 340, respectively). (C) Distribution of centrosomes (red dots) from neurons with an axon in the indicated direction (*n* = 43, 11, 17, and 21 for the L1, L2, L3, and L4 directions, respectively). (D) Directions of neuritic outgrowth represented by vectorial forces showing tensions exerted along each neurite, supposedly all equal in amplitude (stage 2, undifferentiated neurites). The resultant is drawn in red and points down and leftward.
(PDF)

Movie S1 **Neuritic unhookings observed on a DW4 pattern.** Time-lapse recording of neurons plated for 30 hours. Phase contrast images of living cells were captured with a charge-coupled device camera (CoolSNAP HQ; Roper Scientific) using a *20x Phase3 Plan ApoChromat oil NA 1.4* objective mounted on an inverted motorized microscope (Axiovert 200 M; Carl Zeiss, Inc.) equipped with a device enabling regulation of temperature and CO_2_ levels and controlled by MetaMorph software (MDS Analytical Technologies).
(MOV)

Text S1 **Probabilistic Model of Axonal Polarization on DW4 patterns.**
(DOC)
