# Supplementary figures and images for: How Morphological Constraints Affect Axonal Polarity in Mouse Neurons
Source: PLoS One. 2012 Mar 21;7(3):e33623. doi: 10.1371/journal.pone.0033623 (PMC3310070; doi:10.1371/journal.pone.0033623)

**
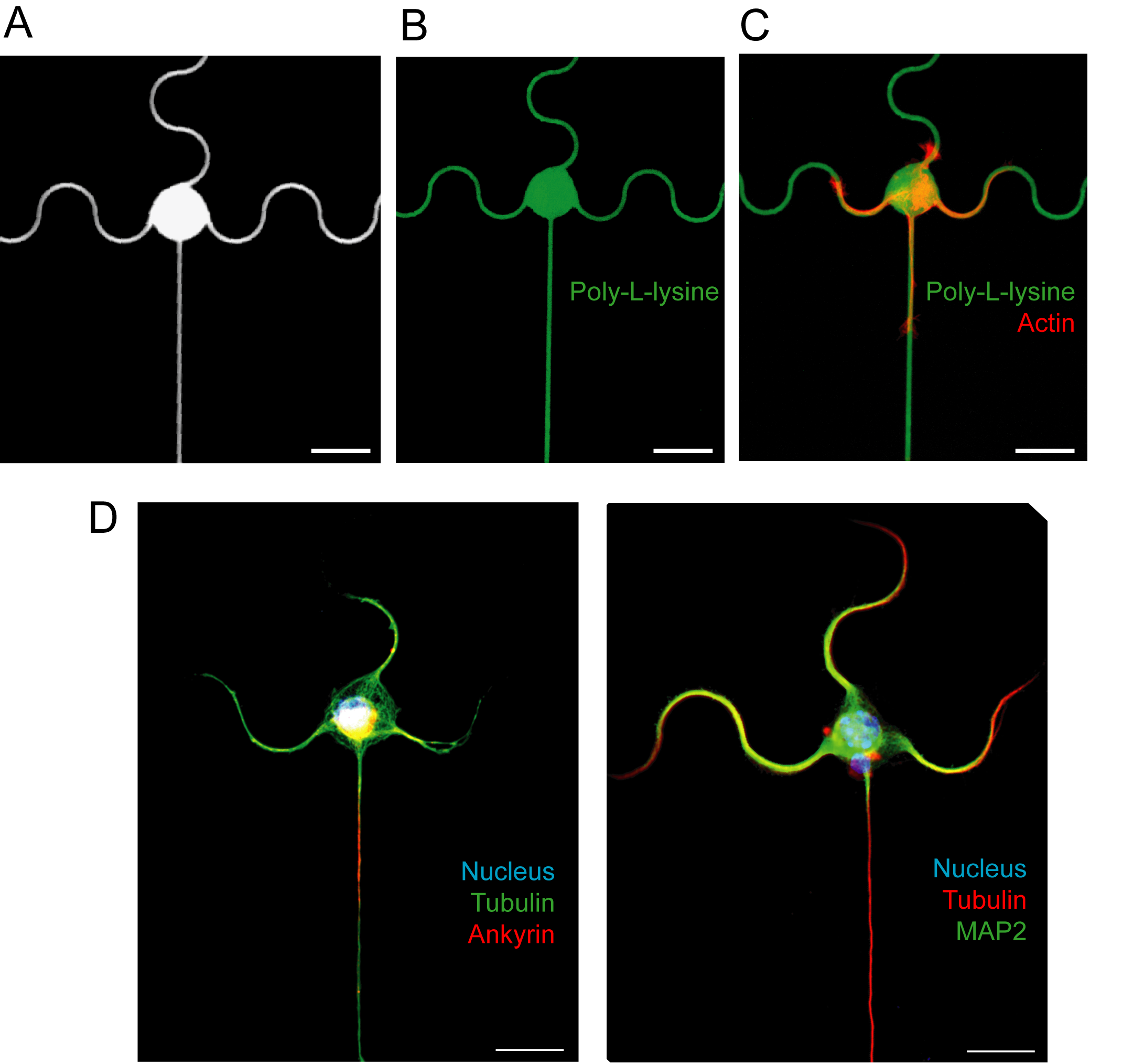
**

**Figure S1**

Supplement: Figure S1 — Micropattern with contrasted adhesiveness. (A) Micrograph of a micropattern showing the mask used during photolithography and properties of the resulting surface: adhesive in white and non adhesive in black. (B) Micrograph of the micropattern showing the adhesive surface covered by FITC-grafted poly-L-lysine (green). (C) Micrograph showing a hippocampal neuron (phalloidin-Texas red staining of actin) spread on the adhesive surface (poly-L-lysine in green). Scale bar 20 µm. (D) Micrographs of hippocampal neurons after 7 days in vitro. (Left) axonal labeling with ankyrin G (red), microtubules (tubulin, green) and nuclei (Hoechst staining, blue); (Right) dendritic marker MAP2 (green), microtubules (tubulin, red) and nuclei (Hoechst staining, blue). Scale bar = 20 µm. (DOC) [file pone.0033623.s001.doc]

**
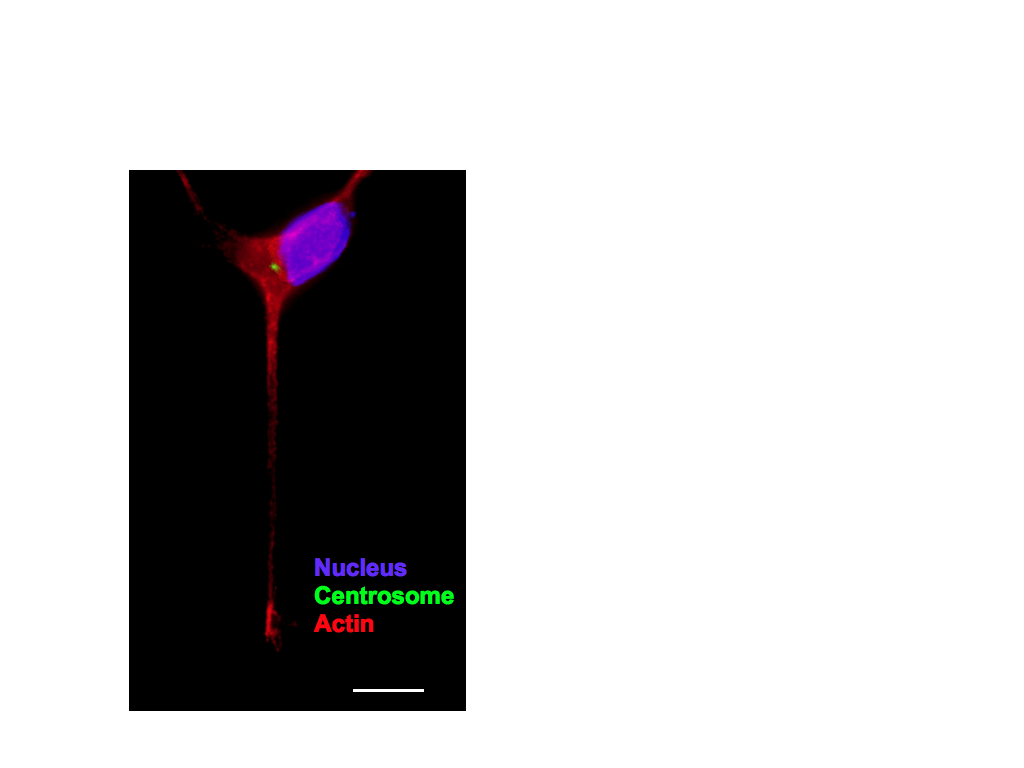
**

**Figure S3**

Supplement: Figure S3 — Actin network of a stage 2 neuron (1 DIV) grown on BmS pattern. Cell nucleus (Hoechst staining, blue), centrosome (γ tubulin labeling, green), and actin (phalloidin-Texas red staining, red). No actin stress fibers are visible around the cell body. Scale bar, 10 µm. (DOC) [file pone.0033623.s003.doc]

**
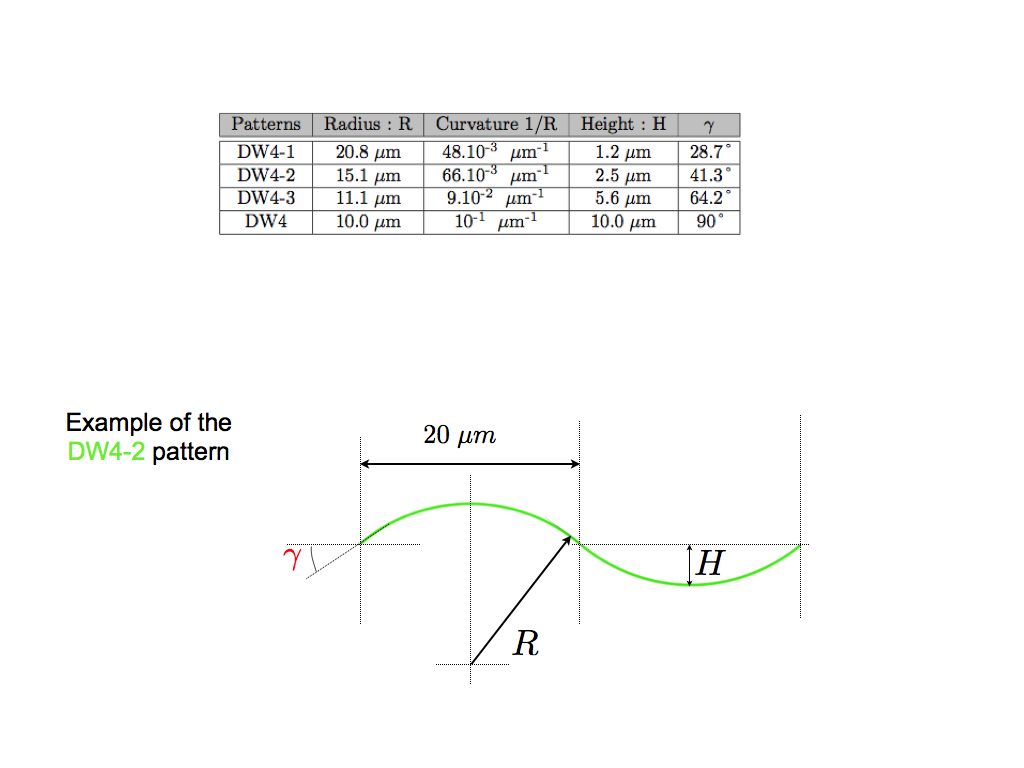
**

**Figure S4**

Supplement: Figure S4 — Summary of geometrical characteristics of DW patterns. (DOC) [file pone.0033623.s004.doc]

**
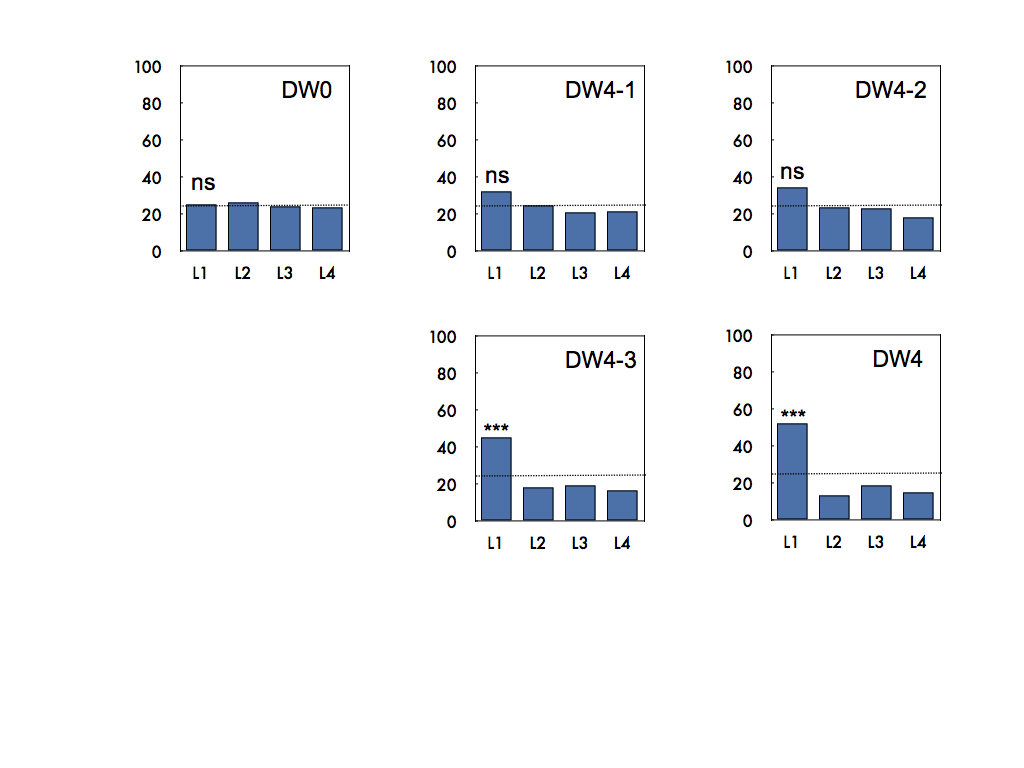
**

**Figure S5**

Supplement: Figure S5 — Influence of neurite curvature on axonal polarization. Axonal specification along the L1–L4 directions was plotted for DW0 and for each DW4 pattern. The number of neurons polarizing along L1 increased with curvature; other neurons polarized uniformly along L2–L4 directions. ***, significantly different from random distribution (dotted line), p<0.001. DW0: 24.4%, 24.4%, 26.0% and 25.2% for L1, L2, L3 and L4, respectively. DW4-1: 32.6%, 24.6%, 21.1% and 21.8% for L1, L2, L3 and L4, respectively. DW4-2: 34.7%, 23.6%, 23.6% and 18.2% for L1, L2, L3 and L4, respectively. DW4-3: 45.5%, 18.2%, 19.7% and 16.7% for L1, L2, L3 and L4, respectively. DW4: 52.3%, 13.3%, 18.8% and 15.1% for L1, L2, L3 and L4, respectively. (DOC) [file pone.0033623.s005.doc]

A

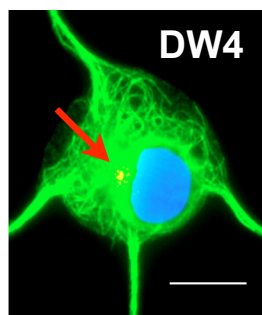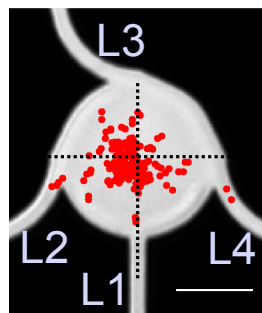

B

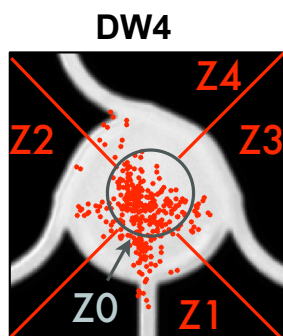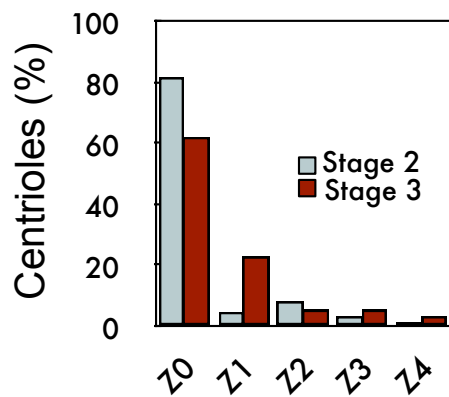

C

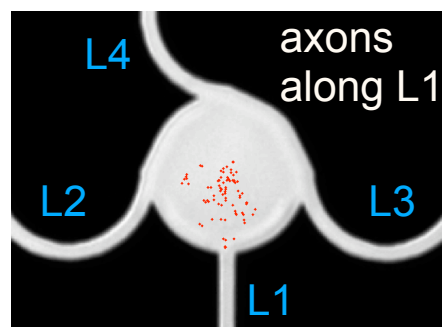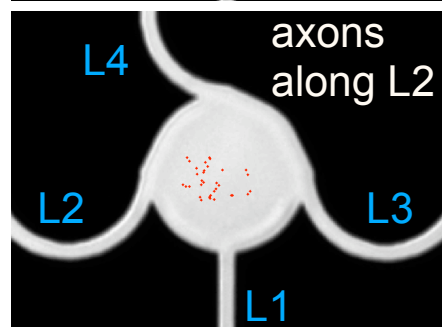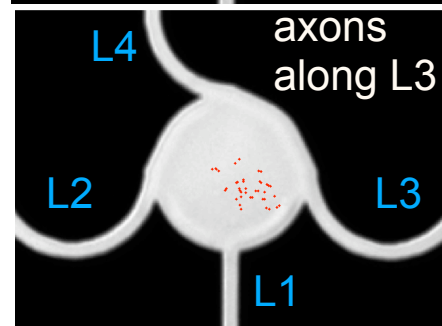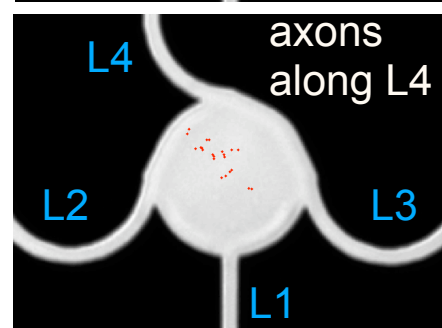

Supplement: Figure S6 — Centrosome distribution and correlation with axonal localization in stages 2 (1 DIV) and 3 (3 DIV) neurons grown over DW4 patterns. (A) Top: an example of microtubule (green), nuclei (blue) and centrosome (red and red arrow) immunolabelings. Scale bar = 10 µm. Bottom: Superimposition of centrosome density map from stage 2 neurons (n = 174) and DW4 micropattern; Scale bar = 10 µm. (B) Top: Scheme of a DW4 pattern and regions of interest Z0–Z4; the scatter plot of centrosome distribution from stage 3 neurons was superimposed (red dots). Bottom: Percentages of centrosomes located in each region of interest from stage 2 and stage 3 neurons (n = 174 and 340, respectively). (C) Distribution of centrosomes (red dots) from neurons with an axon in the indicated direction (n = 43, 11, 17, and 21 for the L1, L2, L3, and L4 directions, respectively). (D) Directions of neuritic outgrowth represented by vectorial forces showing tensions exerted along each neurite, supposedly all equal in amplitude (stage 2, undifferentiated neurites). The resultant is drawn in red and points down and leftward. (PDF) [file pone.0033623.s006.pdf]
