# Supplementary material for: How Morphological Constraints Affect Axonal Polarity in Mouse Neurons
Source: PLoS One. 2012 Mar 21;7(3):e33623. doi: 10.1371/journal.pone.0033623 (PMC3310070; doi:10.1371/journal.pone.0033623)
Supplement: Text S1 — Probabilistic Model of Axonal Polarization on DW4 patterns. (DOC) [file pone.0033623.s008.doc]

**Text S1 Probabilistic Model of Axonal Polarization on DW4 patterns.**

Neurons grown over DW4 micropatterns displayed significant preference for axonal polarization along L1 at 3 DIV but, initially, any of the 4 growing neurites could have differentiated into an axon. The discrepancy between random choice at 1-2 DIV and axonal preference along L1 at 3 DIV corresponds to failures of polarization along curved lines. It is thus possible to calculate a success probability for a neurite to become an axon when growing over a curved line, based on the assumption that 100% of neurites trying to polarize into axons on straight lines actually do.

On DW4 pattern the measured value for polarization along L1 is 52.3% (113 axons along L1 out of 216 neurons). As 113 neurons with an axon along L1 were counted at 3 DIV, as many attempted to differentiate along each direction at 1-2 DIV. Since a mean of 34.3 axons along each of the curved lines (103 axons divided by 3 directions L2-L4) was calculated, the probability to polarize an axon along a curved line is 34.3 actual axons / 113 attempted axons i.e. 30.4%.

The probability of success for attempted polarization along curved lines was then used to predict the preference of axonal polarization along L1 in the presence of drugs inducing the specification of multiple axons. In the presence of cytochalasin D or taxol, after the differentiation of one axon, neurons will try to develop a second axon and will unequally succeed, according to whether they have developed the first axon on the straight line L1 or along any of the curved lines L2-L4.

* Case 1: the first axon was along L1 (52.3 % on DW4). The mean success rate of a second axon polarizing on any other directions would be (3 x 30.4) / 3 = **30.4%.**

* Case 2: the first axon was along any of the curved lines. The mean success rate of a second axon polarizing on either L1 or one of the two other directions would be (2 x 30.4 + 100) / 3 = **53.6%**.

*In conclusion, the neurons polarized along L1 will fail to develop a second axon more often than those polarized along a curved line, thus reinforcing the number of expected neurons with a unique axon along L1.*

Estimation of the axonal preference along L1 for neurons with a unique axon, on DW4, in the presence of

**Cytochalasin D:**

-153 neurons analyzed

-52.3 % polarized on L1 on DW4 pattern in absence of drugs

-78.6 % neurons will attempt to develop multiple axons (MA), as determined on DW0 pattern

| **Cytochalasin**  **Cytochalasin D** | Estimation of the percentage of axonal preference along L1 for neurons with a unique axon | | | | |
| --- | --- | --- | --- | --- | --- |
| *n* = 153 neurons | *Predicted (n)*  [52.3% along L1 without drug on DW4] | *Neurons attempting multiple axons (n):* [78.6 % of *predicted*] =*aMA* | % of success to develop a second axon along the remaining directions | *Successful neurons with multiple axons (n):* [% success *of MA]* | *Expected neurons with a unique axon* [Predicted *(n)* minus sMA *(n)*] |
| Axons along L1 | 80 | 62.88 | 30.4 (along L2-L3-L4) | 19.12 | *n* = 80-19.12  = 60.88 |
| Axons along L2, L3, or L4 (mean) | 24.33  (x3) | 19.12  (x3) | 53.6  (x3) | 10.25  (x3) | *n* = 24.33-10.25  = 14.08 |

-predicted MA neurons: 19.12 + (3 x 10.25) = 49.87 neurons, 32.6% of 153 observed neurons

-neurons with a unique axon: 60.88 + (3 x 14.08) = 103.12 neurons

-predicted axonal preference along L1 for neurons with a unique axon on DW4 in the presence of **CD**: 60.88 / 103.12 = **59.0% (blue dotted line in Fig. 6)**

**Taxol:**

-319 neurons analyzed

-52.3 % polarized on L1 on DW4 pattern in absence of drugs

-73.3 % neurons will attempt to develop multiple axons (MA), as determined on DW0 pattern

| **Taxol**  **Cytochalasin D** | Estimation of the percentage of axonal preference along L1 for neurons with a unique axon | | | | |
| --- | --- | --- | --- | --- | --- |
| *n* = 319 neurons | *Predicted (n)*  [52.3% along L1 without drug on DW4] | *Neurons attempting multiple axon (n):* [73.3 % of *predicted*] =*aMA* | % of success to develop a second axon along the remaining directions | *Successful neurons with multiple axons (n):* [% success *of MA]* | *Expected neurons with a unique axon* [Predicted *(n)* minus sMA *(n)*] |
| Axons along L1 | 166.84 | 122.29 | 30.4 (along L2-L3-L4) | 37.18 | *n* = 166.84-37.18  = 129.66 |
| Axons along L2, L3, or L4 (mean) | 50.72  (x3) | 37.18  (x3) | 53.6  (x3) | 19.93  (x3) | *n* = 50.72-19.93  = 30.79 |

-predicted MA neurons: 37.18 + (3 x 19.93) = 96.97 neurons, 30.4% of 319 observed neurons

-neurons with a unique axon: 129.66 + (3 x 30.79) = 222.03 neurons

-predicted axonal preference along L1 for neurons with a unique axon on DW4, in the presence of **Taxol**: 129.66 / 222.03 = **58.4% (blue dotted line in Fig. 6)**
